# Coverage of tuberculosis and diabetes mellitus screening among household contacts of tuberculosis patients: a household-based cross-sectional survey from Southern Thailand

**DOI:** 10.1186/s12889-020-09090-w

**Published:** 2020-06-18

**Authors:** Myo Minn Oo, Nattaporn Tassanakijpanich, Moe Hnin Phyu, Nanda Safira, Shashi Kandel, Kemmapon Chumchuen, Li Mei Zhang, Hnin Aye Kyu, Porraporn Sriwannawit, Bintinee Bilmumad, Li Cao, Yingwu Guo, Jarawee Sukmanee, Vu Manh Cuong, Virasakdi Chongsuvivatwong, Edward B. McNeil

**Affiliations:** 1 Epidemiology Unit, Faculty of Medicine, Prince of Songkla University, Hat Yai, Thailand; 2 National TB Programme, Department of Public Health, Nay Pi Taw, Myanmar; 3 People’s Hospital of Chuxiong Prefecture, Yunnan, China

**Keywords:** Tuberculosis screening, Household contact investigation, Diabetes screening, Coverage of tuberculosis, Coverage of diabetes, Predictors, Thailand

## Abstract

**Background:**

The comorbid presence of tuberculosis and diabetes mellitus has become an increasingly important public health threat to the prevention and control of both diseases. Thus, household contact investigation may serve a dual purpose of screening for both tuberculosis and diabetes mellitus among household contacts. We therefore aimed to evaluate the coverage of screening for tuberculosis and diabetes mellitus among household contacts of tuberculosis index cases and to determine predictors of tuberculosis screening.

**Methods:**

A household-based survey was conducted in February 2019 in Muang district of Phatthalung Province, Thailand where 95 index tuberculosis patients were newly diagnosed with pulmonary or pleural tuberculosis between October 2017 and September 2018. Household contacts of the index patients were interviewed using a structured questionnaire to ascertain their past-year history of tuberculosis screening and, if appropriate, diabetes mellitus screening. For children, the household head or an adult household member was interviewed as a proxy. Coverage of tuberculosis screening at the household level was regarded as households having all contacts screened for tuberculosis. Logistic regression and mixed-effects logistic regression models were used to determine predictors of tuberculosis screening at the household and individual levels, respectively, with the strengths of association presented as adjusted odds ratios (AOR) and 95% confidence intervals (CI).

**Results:**

Of 61 responding households (64%), complete coverage of tuberculosis screening at the household level was 34.4% and among the 174 household contacts was 46.6%. About 20% of contacts did not receive any recommendation for tuberculosis screening. Households were more likely to have all members screened for tuberculosis if they were advised to be screened by a healthcare professional rather than someone else. At the individual level, contacts aged ≥35 years (AOR: 30.6, 95% CI: 2.0–466.0), being an employee (AOR: 0.1, 95% CI: 0.0–0.8) and those who had lived more than 5 years in the same household (AOR: 0.1, 95% CI: 0.0–0.8) were independent predictors for tuberculosis screening. Coverage of diabetes mellitus screening was 80.6% with lack of awareness being the main reason for not being screened.

**Conclusions:**

Compared to diabetes screening, the coverage of tuberculosis screening was low. A better strategy to improve coverage of tuberculosis contact screening is needed.

## Background

Globally around 3 million new tuberculosis cases remain undiagnosed [1]. Systematic evaluation for tuberculosis among high-risk groups produces a high yield of detection, hence reducing its transmission in the community [2, 3]. Approximately 3% of household contacts were reported to have active disease at the time of their household members’ diagnosis [4]. Household contact investigation is one of the recommended strategies to enhance tuberculosis case detection in high burden countries [5]. However, this practice has been not been implemented well in most resource-limited countries [6]. Thailand is one of the 30 highest burden countries in the world, with an estimated incidence of 172 per 100,000 population [1]. Of 108,000 estimated cases in Thailand, only 76% were notified in 2018, which indicates a substantial gap in case detection.

A study from Uganda indicated that only 50% of household contacts were investigated for tuberculosis by health workers [7]. A study from India reported that 20% of household contacts refused to participate in investigations for tuberculosis [8]. In Brazil, 29% of household contacts of adults with newly diagnosed tuberculosis were not evaluated with 62% of them being smear-positive [9]. In Bangkok, Thailand, only 52% of the eligible cases brought their household contacts to the clinic for further investigation [10]. Younger age, male sex [11], low income, and low education of household heads [12] were reported to be associated with low coverage of household contact investigation and found to be clustered among each household. A high level of perceived susceptibility, low level of perceived barriers, high intention to bring their contacts, and shorter distance from home to the tuberculosis clinic all influence the completion of such investigations [10].

The comorbid presence of tuberculosis and diabetes mellitus (DM) is an increasingly important public health threat to the prevention and control of both diseases [13–15]. Due to their immune dysfunction, people with DM are more likely to contract a new tuberculosis infection and/or disease reactivation, develop active disease, and experience poor treatment outcomes [16]. Moreover, the prevalence of DM is gaining momentum in developing countries, adding an additional burden [17, 18]. A recent study from India reported an alarmingly high proportion of household contacts with diabetes or pre-diabetes at nearly 40% [19]. This synergetic relationship between tuberculosis and diabetes has thus prompted WHO to publish a provisional collaborative framework for the care and control of both diseases [20]. Hence, household contact investigation can serve a dual purpose of screening for both tuberculosis and DM among household contacts [21]. Among Southeast Asian countries, Thailand has one of the highest prevalence of diabetes [17].

In Thailand, routine investigation for tuberculosis among household contacts of known index cases is one of the active case-finding strategies. This is conducted by public health officers in collaboration with community volunteers. Annual screening for diabetes mellitus is conducted among those aged 35 and above with a fasting blood sugar test [22].

In Thailand, control of tuberculosis at the national level is overseen by the Bureau of Tuberculosis under the Ministry of Public Health in collaboration with other non-ministerial institutions and agencies. The public health system is also aligned with hospitals that help to collaborate in conducting household contact investigations.

When a patient is diagnosed with tuberculosis at the hospital, a responsible nurse educates the index case or caregiver about the importance of tuberculosis screening for their household contacts and asks them to bring all other household members to the hospital to have a chest radiograph. After discharge from hospital, the index case is transferred to the sub-district health promotion hospital to continue their medication. If any household contact fails to adhere to the screening process, a public health officer will inform them or their caregiver to do so at that hospital. Public health officers and community health volunteers routinely visit the household of all index cases, provide counseling, and recommend other household members to be screened for tuberculosis. Screening is done with chest radiography, and if positive or suggestive of tuberculosis, then other confirmatory investigations are done.

There is limited evidence on the coverage of these investigations and screening among household contacts of index tuberculosis patients. Early screening among high-risk populations such as household contacts provides a critical and unique window of opportunity for public health program interventions. Therefore, this study aimed to assess the coverage of tuberculosis and diabetes mellitus screening and predictors of tuberculosis screening among household contacts of index tuberculosis patients in Phatthalung Province, an area in southern Thailand with a high prevalence of both diseases. It is hoped that the results of this study may provide information about the ground situation on household contact investigations conducted in a rural area of southern Thailand. Furthermore, information on diabetes mellitus screening will also provide a useful insight into the prevention and control of tuberculosis and diabetes mellitus in Thailand.

## Methods

### Study design and setting

**Fig. 1:**
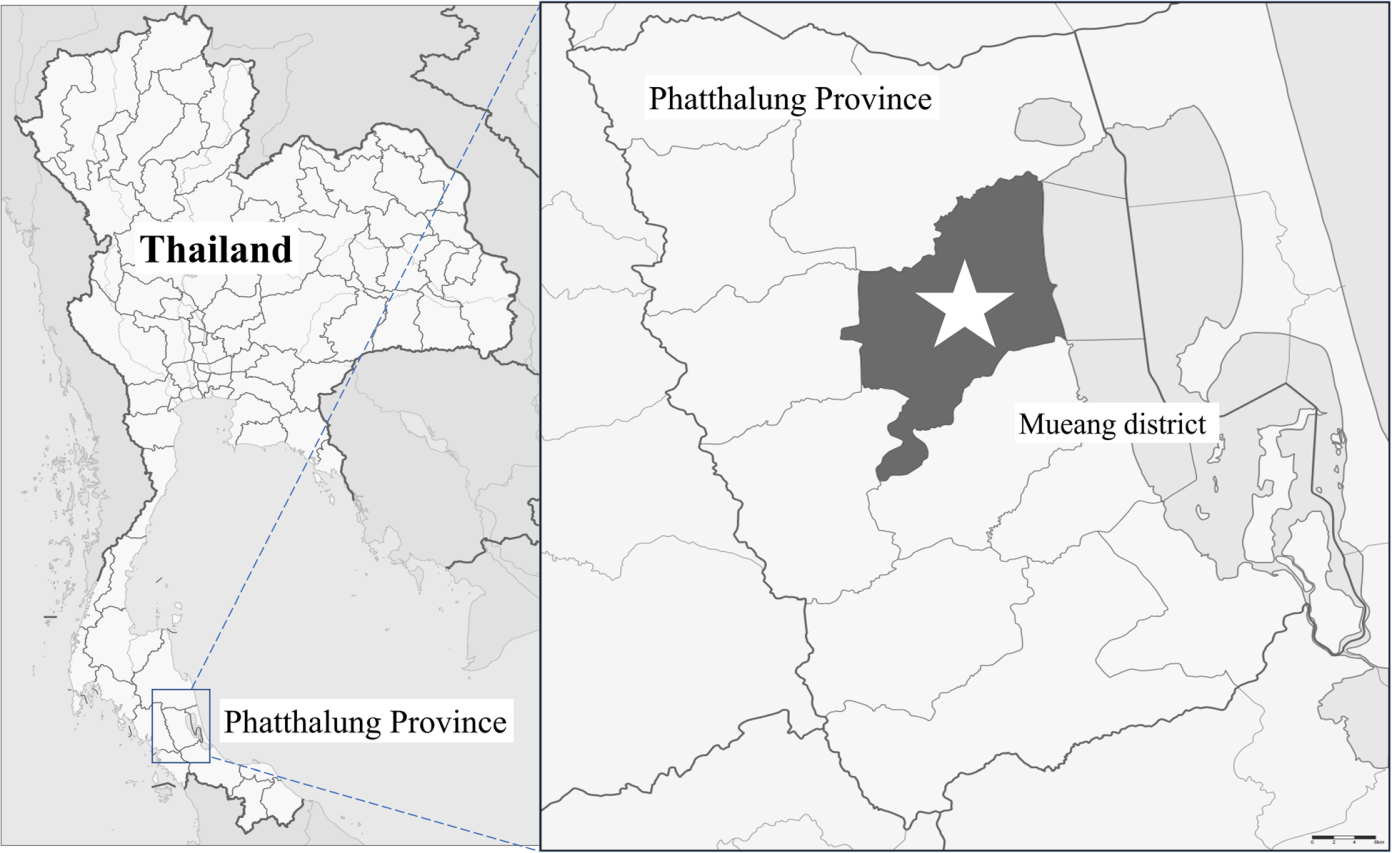
Map showing Muang district (starred) in Phatthalung Province, Southern Thailand. *Source: Created using R software. GNU General Public License, version 3*

A household-based cross-sectional survey was conducted during February 2019 in Muang district of Phatthalung Province. Figure 1 shows a map showing the location of the study area in the southern region of Thailand. With a population of 520,419 in 2014, Phatthalung covers an area of around 3424 km^2^ and is 860 km from Bangkok, the capital city of Thailand. The district is divided into 14 sub-districts which are comprised of 144 villages.

### Study participants

A household contact was defined as a person who shared the same enclosed living space for one or more nights or frequent or extended periods during the day with an index tuberculosis case during the 3 months before the commencement of the current treatment episode [5]. Index patients were all newly diagnosed with pulmonary or pleural tuberculosis between October 2017 and September 2018 in Phatthalung hospital. We aimed to recruit all household contacts of these index patients (for the assessment of tuberculosis screening coverage) and all household contacts aged ≥35 years (for the assessment of diabetes screening coverage).

### Sample size

According to the Phatthalung provincial health office, the coverage of tuberculosis screening among household contacts was estimated to be around 83% and that of DM screening among the general population aged 35 years or above at more than 90%. Estimating the true prevalence with a precision of ± 5% and allowing for a 10% non-response rate using the following sample size formula for estimating a single proportion would require a minimum sample of 239 household contacts.

$$ n={\left(\frac{Z_{\alpha }}{d}\right)}^2\times \hat{p}\left(1-\hat{p}\right), $$

where Z_α_ is the standard normal deviate at level α (0.05), $\hat{p}$ is the estimated coverage of tuberculosis or DM screening, and *d* is the precision of this estimate (5%).

### Data collection

Data were collected from the medical records of all index patients and their household contacts using a pilot-tested structured questionnaire modified from Lung et al. [23] A list of index tuberculosis patients and their baseline demographic characteristics (age, sex, and employment status) and clinical information (tuberculosis type, treatment, smear result, history of chronic kidney disease, diabetes mellitus, hypertension, and HIV status) were obtained from the Phatthalung Provincial Health Office. All data collectors were trained for consistency in data collection. Community health volunteers assigned by the health office contacted index cases and/or heads of their households at least 2 weeks before the study commenced, informed them about the study, and requested permission for a researcher to visit their household. Those who agreed to be interviewed were visited by the researchers during February 2019. The researchers introduced themselves and obtained verbal informed consent from each household member. For children or those who were unable to be interviewed, researchers interviewed their guardians or caregivers instead. The interview guideline is shown in the supplementary file.

All eligible household contacts who were present at the time of the visit were interviewed face-to-face by the trained researchers using the questionnaire. The following data were obtained: household-level information such as type of dwelling, type of construction materials of dwelling, ownership of any commodity items (car, motorbike, television, radio, mobile phone, personal computer), number of bedrooms in the household, type of person giving recommendation to the household for tuberculosis screening and time interval since the index diagnosis, accessibility to screening program (distance to nearest health facility from home, type, and cost of transportation used to access care, waiting time at nearest hospital and rating of the overall healthcare service); and household contact level information such as socio-demographic characteristics (age, sex, education, occupation, relationship with the index case, and duration of time living in the household). Information on any chest X-ray or sputum examination and skin test for children during the past 12 months for coverage of tuberculosis screening were obtained as well as information on DM and fasting blood sugar test for DM screening during the past 12 months. To minimize recall bias, pictorial aids of tuberculosis screening (a person having a chest X-ray and inoculating tuberculin under the skin) were also used.

For children aged 5 to 18 years, if tuberculosis infection is not suspected, either the tuberculin skin test (TST) or interferon-gamma release assays (IGRA) will usually be performed. Prophylactic medication is considered according to the test result. All children who are less than 5 years old or have immunodeficiency have a high risk of tuberculosis infection, therefore, prophylactic medication has to be prescribed without the need to perform TST or IGRA.

### Statistical analysis

The data were double-entered into EpiData version 3.1, validated, and then corrected for any inconsistencies. R version 3.2.1 was used for all statistical analyses. Descriptive statistics were used to summarize characteristics of households and index tuberculosis patients with frequencies and percentages for categorical variables and means and standard deviations or medians and interquartile ranges for continuous variables as appropriate. A household was considered to be screened for tuberculosis if all contacts were screened. Logistic regression modeling was used to determine predictors of tuberculosis screening at the household level. To determine predictors at the individual level, mixed-effects logistic regression models were constructed using the household as the random slope to account for the hierarchical nature of the data. Variables were selected into the model by comparing models with different variables using the chi-squared goodness-of-fit test. Multi-collinearity was also assessed and any variables with significant collinearity were removed from the model. A two-sided *p*-value of < 0.05 was considered statistically significant.

## Results

**Fig. 2:**
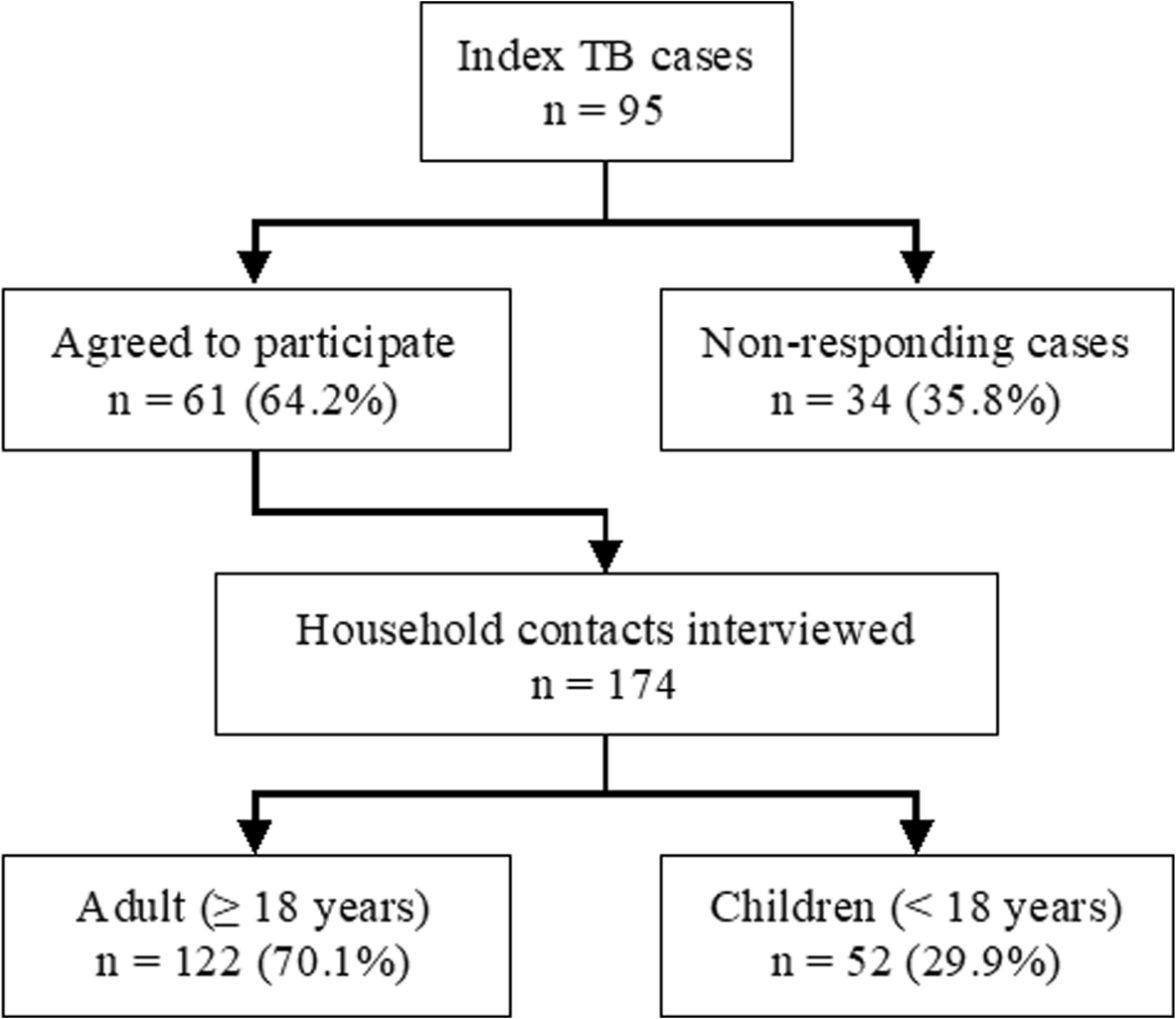
Participant flow diagram of the household survey

Of 95 index tuberculosis cases registered in Phatthalung hospital during the previous aforementioned 12-month period, the heads of 61 households agreed to participate in the study (response rate 64.2%). As shown in Fig. 2, a total of 174 household contacts were interviewed: 122 adults (≥ 18 years of age) and 52 children (< 18 years of age).

### Characteristics of index tuberculosis patients

About 70% of index tuberculosis patients were male, and their median (interquartile range) age was 50 (38–64) years. The employment rate among the 60 adult-aged index cases was high at 75%. Most had pulmonary tuberculosis and had received their initial treatment regimen. Nearly 30% had at least one co-existing condition such as chronic kidney disease, diabetes mellitus, hypertension or HIV infection. Nearly half (48%) were smear-positive. Comparison of index tuberculosis patients between responding and non-responding households showed that index patients whose households participated in the study were, on average, 8 years older than those whose households refused to participate (*p*-value < 0.05). No other factors were significantly different between responding and non-responding households (supplementary file S1).

### Coverage and predictors of tuberculosis screening at the household level

Overall, almost 20% of households did not receive any screening recommendation. Almost half (49%) received a recommendation within 1 month of the index case’s diagnosis, about one fifth received a recommendation between 2 and 6 months after diagnosis, and about 5% received a recommendation more than 6 months after diagnosis.

Of the 61 households that responded, 21 (34.4%) reported that all of their household contacts had been screened for tuberculosis during the past 12 months. Characteristics of households and their relationship with coverage of tuberculosis screening are shown in Table 1. Of 34 households that had at least one child, 11 (32.4%) reported completing the screening process. Households that were recommended by a health care provider to have their contacts screened for tuberculosis were more likely to have all of their contacts complete screened (AOR = 7.2, 95% CI = 1.3–67.0). No other household-level factor was associated with completing tuberculosis screening.

**Table 1:** Coverage and predictors of tuberculosis screening at the household level in Phatthalung province, Thailand

Characteristic	Sub-category	Total n (%)	Screened n (%)	Crude OR (95% CI)	***p***-value	Adjusted OR (95% CI)	***p***-value
Total		61 (100.0)	21 (34.4)	–	–	–	–
Type of dwelling	Detached	55 (90.2)	16 (29.1)	Ref	–	Ref	–
	Others	6 (9.8)	5 (83.3)	12.2 (1.8–243.5)	0.028	12.6 (1.4–348.8)	0.054
Type of construction material of dwelling	Cement	10 (16.4)	3 (30.0)	Ref	–	–	–
	Concrete	44 (72.1)	15 (34.1)	1.2 (0.3–6.2)	0.805	–	–
	Wood	7 (11.5)	3 (42.9)	1.8 (0.2–14.2)	0.587	–	–
Number of bedrooms in the house	1	9 (14.8)	2 (22.2)	Ref	–	–	–
	2	29 (47.5)	12 (41.4)	2.5 (0.5–18.6)	0.307	–	–
	3+	23 (37.7)	7 (30.4)	1.5 (0.3–12.0)	0.644	–	–
Proxy score for SES^a^	median (IQR)	6 (5–7)	6 (5–7)	1.2 (0.8–1.9)	0.347	–	–
Person from whom household received screening information	HCP	32 (52.5)	14 (43.8)	6.2 (1.4–43.6)	0.028	7.2 (1.3–67.0)	0.040
	Index case and HCP	11 (18.0)	5 (45.5)	6.7 (1.1–56.5)	0.922	6.0 (0.8–64.9)	0.098
	Others^b^	18 (29.5)	2 (11.1)	Ref	–	Ref	–
Time of receiving first recommendation to screen for TB since diagnosis of index patient (months)	≤ 1	30 (49.2)	12 (40.0)	Ref	–	–	–
	2–6	12 (19.7)	5 (41.7)	1.1 (0.3–4.2)	0.921	–	–
	7+	3 (4.9)	1 (33.3)	0.8 (0.0–8.7)	0.822	–	–
Distance to the nearest health facilities (km)	≤ 5	19 (31.1)	8 (42.1)	Ref	–	–	–
	5–10	24 (39.3)	7 (29.2)	0.6 (0.2–2.0)	0.379	–	–
	11+	17 (27.9)	6 (35.3)	0.8 (0.2–2.9)	0.676	–	–
Transportation used to get to the facilities	Owned cars	20 (32.8)	9 (45.0)	Ref	–	Ref	–
	Motorbike	31 (50.8)	11 (35.5)	0.7 (0.2–2.1)	0.498	0.3 (0.0–1.5)	0.163
	Others	9 (14.8)	1 (11.1)	0.2 (0.0–1.1)	0.103	0.1 (0.0–0.9)	0.092
Cost for transportation (Baht)	≤ 50	32 (52.5)	14 (43.8)	Ref	–	Ref	–
	> 50	28 (45.9)	7 (25.0)	0.4 (0.1–1.3)	0.133	0.2 (0.0–1.3)	0.125
Waiting hours at the health facility (hours)	≤ 2	29 (47.5)	12 (41.4)	Ref	–	–	–
	> 2	30 (49.2)	9 (30.0)	0.6 (0.2–1.8)	0.363	–	–
Satisfaction with health services	Very dissatisfied	0 (0.0)	0 (0.0)	–	–	–	–
	Dissatisfied	3 (4.9)	1 (33.3)	1.2 (0.0–17.9)	0.913	–	–
	Neutral	10 (16.4)	3 (30.0)	Ref	–	–	–
	Satisfied	27 (44.3)	9 (33.3)	1.2 (0.3–6.4)	0.848	–	–
	Very satisfied	19 (31.1)	8 (42.1)	1.7 (0.3–9.8)	0.525	–	–
Household with at least one child	No	27 (44.3)	10 (37.0)	0.9 (0.5–1.6)	0.792	–	–
	Yes	34 (55.7)	11 (32.4)	Ref	–	–	–
Number of contacts per household	1	11 (18.0)	5 (45.5)	Ref	–	–	–
	2	18 (29.5)	6 (33.3)	0.6 (0.1–2.8)	0.515	–	–
	3	16 (26.2)	5 (31.2)	0.5 (0.1–2.7)	0.455	–	–
	4+	16 (26.2)	5 (31.2)	0.5 (0.1–2.7)	0.455	–	–

*TB* tuberculosis, *OR* odds ratio, *95% CI* 95% confidence interval, *SES* socioeconomic status, *IQR* interquartile range, *HCP* health care provider, *km* kilometer;

^a^ Based on seven household-owned commodity items: car, motorbike, washing machine, refrigerator, television set, computer, and phone

^b^ Others included community health volunteers and staffs from the health improvement program

### Coverage and predictors of tuberculosis screening at the household contact level

Characteristics of household contacts and tuberculosis screening data are shown in Table 2. Of 174 contacts residing in 61 households, 81 (46.6%) were screened during the past 12 months. Nearly one-third (29.9%) of contacts were less than 18 years of age. Around two-thirds were first degree relatives of the index case and the majority reported that they lived more than 5 years in the same household. Household contacts aged ≥35 years, being an employee, and those who had lived for more than 5 years in the same household were independent predictors for tuberculosis screening.

**Table 2:** Coverage of tuberculosis screening and its predictors at the individual household contact level in Phatthalung province, Thailand

Characteristic	Sub-category	Total n (%)	Screened n (%)	Crude OR (95% CI)	***p***-value	Adjusted OR^a^ (95% CI)	***p***-value
Total		174 (100.0)	81 (46.6)				
Age groups (years)	< 18	52 (29.9)	20 (38.5)	Ref		Ref	
	18–34	27 (15.5)	12 (44.4)	0.8 (0.1–5.2)	0.819	2.7 (0.2–41.1)	0.468
	35+	95 (54.6)	49 (51.6)	5.3 (1.4–20.4)	0.016	**30.6 (2.0–466.0)**	**0.014**
Sex	Male	56 (32.2)	25 (44.6)	0.5 (0.2–1.3)	0.143	–	–
	Female	118 (67.8)	56 (47.5)	Ref		–	–
Education	None	16 (9.2)	6 (37.5)	Ref		–	–
	Primary	73 (42.0)	32 (43.8)	1.6 (0.2–11.2)	0.625	–	–
	Secondary	18 (10.3)	7 (38.9)	0.7 (0.1–7.1)	0.763	–	–
	High	27 (15.5)	12 (44.4)	1.4 (0.2–12.8)	0.754	–	–
	Vocational or non-formal	18 (10.3)	10 (55.6)	2.3 (0.2–24.5)	0.498	–	–
	Bachelor+	22 (12.6)	14 (63.6)	8.7 (0.7–108.9)	0.094	–	–
Occupation	Unemployed	87 (50.0)	40 (46.0)	Ref		Ref	
	Employed	36 (20.7)	14 (38.9)	0.4 (0.1–1.7)	0.220	**0.1 (0.0–0.8)**	**0.034**
	Business owner	25 (14.4)	12 (48.0)	1.9 (0.4–9.5)	0.432	0.4 (0.0–4.2)	0.443
	Officer	26 (14.9)	15 (57.7)	2.9 (0.6–14.3)	0.203	0.8 (0.1–9.4)	0.870
Relationship with index case	Parent	19 (10.9)	10 (52.6)	Ref		–	–
	Spouse	39 (22.4)	23 (59.0)	1.6 (0.1–20.7)	0.704	–	–
	Offspring	53 (30.5)	27 (50.9)	0.4 (0.0–4.5)	0.490	–	–
	Others	63 (36.2)	21 (33.3)	0.0 (0.0–0.5)	0.012	–	–
Sharing the same bedroom with index case	Yes	33 (19.0)	19 (57.6)	Ref		–	–
	No	141 (81.0)	62 (44.0)	0.2 (0.0–0.9)	0.039	–	–
Duration of living in the same household (years)	< 5	30 (17.2)	19 (63.3)	Ref		Ref	
	≥ 5	139 (79.9)	62 (44.6)	0.2 (0.0–1.1)	0.066	**0.1 (0.0–0.8)**	**0.036**
	No Data	5 (2.9)	0 (0.0)	–	–	–	–
Frequency of contacts with index case	Daily	152 (87.4)	76 (50.0)	Ref		–	–
	More than daily	22 (12.6)	5 (22.7)	0.0 (0.0–0.4)	0.005	–	–

*TB* tuberculosis, *OR* odds ratio, *95% CI* 95% confidence interval;

^a^ Due to multicollinearity and small number of observations, two covariates, relationship with index case and frequency of contacts with index case were removed from the final model

### Coverage of diabetes mellitus screening

Among the 174 household contacts, 95 were aged 35 years or more, of which 23 were diagnosed with diabetes mellitus and were currently undergoing treatment. Among the 72 contacts without diabetes, 58 (80.6%) had been screened. Of the remaining 14 contacts, 10 gave reasons for not being screened: six reported that they did not think it was necessary to do so, two did not have time and two were unaware that they needed to be screened.

## Discussion

Coverage of tuberculosis screening was relatively low but coverage of diabetes screening was high in household contacts of index tuberculosis patients. Age ≥ 35 years of age, being an employee, and living in the same household for more than 5 years were independent predictors for tuberculosis screening among household contacts.

Possible reasons for the low coverage for tuberculosis screening may be due to lack of time, a misperception about the need for tuberculosis screening among households of index cases with negative sputum smear examination result or extra-pulmonary tuberculosis, and the high cost of screening (3800 baht, ~US$120) since investigations for tuberculosis are not provided free of charge. This finding is consistent with previous studies in Thailand and other countries [7, 9–11]. However, even lower rates of adherence to tuberculosis screening by household contacts were reported in India [8] and Ethiopia. [24] Three factors (older age, being employed, and duration of living in the same household) were not consistent with other studies. [10–12, 24] This inconsistency could be due to geographic or cultural differences of the study sample or simply due to differences in study methodologies.

The coverage of tuberculosis screening among children was relatively lower than that among adults. Children are in fact at high risk if exposed to adults with smear-positive pulmonary tuberculosis [25, 26]. This risk also increases with the frequency and degree of contact. They also can be an important source of transmission as school students who share a classroom with an infected student have an 8-fold increased risk of tuberculosis infection [27]. This suggests that more effort should be given to encourage screening for children, one of the high-risk groups for contracting tuberculosis from index cases [28].

A high proportion of households did not receive any messages or instructions for their contacts to be screened for tuberculosis despite the main person for such communication being a health care provider and/or index case per se. This may reflect miscommunication or a gap between index cases or health care workers and their household contacts. Stigma surrounding tuberculosis in a closely-knit community may have also contributed. However, the majority of those who did receive such a recommendation received it within 1 month after diagnosis of the index case. This in turn reflects the timeliness of providing recommendations from the provincial health office.

More than half of the household contacts in this study did not receive tuberculosis screening at all. A previous study revealed that a lower perception of barriers and a higher perception of being susceptible to tuberculosis were associated with high coverage of contact screening [10]. However, this emphasizes the fact that some people are still left behind despite the best efforts of the public health system. Hence, there is an opportunity to improve and widen the health information network to reach all these people.

Our study showed that coverage of diabetes screening was relatively high among household contacts and was higher than the number reported in the general Thai population which was around 56% in 2018 (data provided by the Phatthalung Provincial Health Office). However, the characteristics of household contacts and those of the general population may be very different.

The strengths of the study were addressing research questions of both tuberculosis and diabetes screening in the one study, selection of a rural area where the public health care system may be weaker than in urban areas, and full cooperation from the local public health office which is important for advocating policy and practical changes.

Limitations of this study relate to the design and operational nature of the study; the low response rate, the consequent small individual-level sample size for multivariate analysis, and recall bias due to the self-reported nature of the data. The low response rate from the household is likely due to several reasons. Although appointments were made with household heads 2 weeks in advance, some refused at the time of the interview, probably due to inconvenience or stigma. In some cases, the index case had not disclosed their disease status to other members of the household. Other possible explanations for the low response rate include lack of time due to busy schedules or sudden emergencies, change of mind, and forgetfulness. Our reported screening coverage may therefore be underestimated.

All data were self-reported and there was no validation with other data sources such as registers from the hospital outpatient department. Thus, under- or over-reporting may be an issue. We were also unable to qualitatively explore the reasons for failing to complete the screening process for tuberculosis and diabetes mellitus. Second, due to the low response rate, the power to detect significant predictors of tuberculosis screening was low. Finally, since all information related to screening was self-reported, this may contribute to some level of recall bias. However, to minimize this, we limited the timeframe to the past 12 months and used pictorial aids during the interviews. All these points subsequently limit the generalizability of our study.

There are two critical programmatic implications from this study. First, coverage of tuberculosis screening needs to be as close to 100% as possible. In order to achieve this, it is essential to ensure that the recommendation for tuberculosis screening must reach all household members through index cases and/or healthcare providers. Strategies such as opening clinics outside office or working hours should be used for those household members working full-time or who are too busy to attend the screening process during clinic opening times. Other innovative tracing methods such as sending persistent reminders via mobile phone text messages, phone calls, or even internet calls should be considered to improve compliance.

Second, although our study reported a relatively high level of coverage for diabetes screening, there is still room for improvement. Thailand has already implemented annual routine diabetes checkups among the general population aged 35 years or more. Since household contacts themselves have a higher risk of contracting tuberculosis, a hidden diabetes condition might increase the risk of infection. Hence, it is crucial to screen all eligible household contacts for diabetes. Our study highlighted that accessing diabetes information through household contact investigation is possible and is a golden opportunity for dual public health implementation. Hence, local provincial health offices should consider taking “the number of contacts ≥ 35 years of age screened for diabetes” as a routine indicator in their programmatic monitoring system.

## Conclusions

Although the coverage of tuberculosis screening was low, it could be improved by better communication of service recommendations and tracing strategies in place at the public health office. This would not only be important for affected households, but it would also prevent further transmission of infection within the community.

## Supplementary information

**Additional file 1.** The file “Interview Guide “ contains information for the interviewers during the interviews with participants.

## Data Availability

Data can be obtained from the corresponding author under reasonable request.

## Abbreviations

DM: Diabetes Mellitus
TB: Tuberculosis
WHO: World Health Organization
