# Supplementary material for: Coverage of tuberculosis and diabetes mellitus screening among household contacts of tuberculosis patients: a household-based cross-sectional survey from Southern Thailand
Source: BMC Public Health. 2020 Jun 18;20:957. doi: 10.1186/s12889-020-09090-w (PMC7301490; doi:10.1186/s12889-020-09090-w)
Supplement: Supplementary file 1 — Additional file 1. The file “Interview Guide “ contains information for the interviewers during the interviews with participants. [file 12889_2020_9090_MOESM1_ESM.docx]

**Supplementary file**

**Interview Guide**

**Coverage of tuberculosis and diabetes mellitus screening among household contacts of
tuberculosis patients: a household-based cross-sectional survey from Southern Thailand**

Objectives

1. To assess the coverage of tuberculosis and diabetes screening
2. To assess barriers of receiving tuberculosis and diabetes screening

Guide

1. The interviewer explains the purpose of the research to the participants and provides the participant information sheet.
2. The interviewer explains the composition of the questionnaire, which consists of 4 main topics: general information, understanding of tuberculosis, coverage of diabetes and tuberculosis screening, and attitudes to tuberculosis prevention and screening and access to screening of people exposed to tuberculosis in households.
3. Interviewer asks for verbal consent to join the study and conducts the face-to-face interview.

Approximate duration for interviews should be no more than 30 minutes
